# Adapting the local response for malaria elimination through evaluation of the 1-3-7 system performance in the China–Myanmar border region

**DOI:** 10.1186/s12936-017-1707-1

**Published:** 2017-01-31

**Authors:** Duoquan Wang, Chris Cotter, Xiaodong Sun, Adam Bennett, Roly D. Gosling, Ning Xiao

**Affiliations:** 1 National Institute of Parasitic Diseases, Chinese Center for Disease Control and Prevention, Key Laboratory of Parasite and Vector Pathology, World Health Organization Collaborating Centre for Tropical Diseases, National Center for International Research on Tropical Diseases, Shanghai, 200025 People’s Republic of China; 2 Malaria Elimination Initiative, Global Health Group, University of California, San Francisco, San Francisco, CA 94158 USA; 3 Yunnan Institute of Parasitic Diseases, Puer, 665000 People’s Republic of China

**Keywords:** Malaria elimination, China–Myanmar border, 1-3-7 Strategy, Reactive case detection, Evaluation

## Abstract

**Background:**

Assessing the essential components of ‘1-3-7’ strategy along the China–Myanmar border is critical to identify gaps and challenges to support evidence-based decision making.

**Methods:**

A mixed-method retrospective study including quantitative and qualitative analysis of the 1-3-7 system components was conducted. Sampled counties were chosen based on malaria incidence from 1 January 2012 to 31 December 2014.

**Results:**

All 260 confirmed malaria cases from sampled counties were reported within 1 day and had completed case investigations. 70.0% of all Reactive Case Detection (RACD) events were conducted and 90.1% of those were within 7 days. Only ten additional individuals were found malaria positive out of 3662 individuals tested (0.3%) by rapid diagnostic test during RACD events.

**Conclusions:**

Key gaps were identified in case investigation and RACD activities in Yunnan Province border counties. This evidence supports improving the RACD (or “7”) response strategy in this setting. Given the challenges in this border region, it will be critical to adapt the RACD response to promote the malaria elimination along the China border.

**Electronic supplementary material:**

The online version of this article (doi:10.1186/s12936-017-1707-1) contains supplementary material, which is available to authorized users.

## Background

 A strong political commitment was made by the National Health and Family Planning Commission of the People’s Republic of China (former Ministry of Health) by issuing the National Malaria Elimination Action Plan (NMEAP; 2010–2020) in 2010. The goal of the NMEAP is to eliminate locally acquired malaria by the end of 2015, except for the bordering areas in Yunnan Province, and to interrupt local malaria transmission by 2020 nationwide [1]. The National Malaria Elimination Programme (NMEP) strategy in China is a surveillance and response system for case reporting, investigation, and follow-up response known as ‘1-3-7’: reporting of malaria cases within 1 day, case confirmation and investigation within 3 days, and appropriate foci response to prevent potential local transmission within 7 days [2]. The ‘1-3-7’ strategy was rolled out nationally in 2012 and is an important and simple set of targets for local Centers for Disease Control and Prevention at the country level to follow.

Surveillance and response is the most important component driving the NMEP as the efficient response to individual cases and the foci treatment are vital strategic measures in eliminating malaria transmission [3, 4]. As malaria transmission declines, strategies for detecting and targeting clusters of infection, whether geographic or demographic, become important to reduce the local parasite reservoir [5]. Many countries are currently implementing a reactive case detection (RACD) strategy to achieve and maintain malaria elimination [6–10]. RACD targets spatial and temporal factors associated with finding infections and involves testing symptomatic and asymptomatic individuals residing in a specified area, typically a pre-determined radius around locally acquired or imported cases with potential transmission risk [11, 12].

Previous assessments of the ‘1-3-7’ targets identified gaps in reporting in the malaria information system, particularly in Yunnan Province [13, 14]. Furthermore, recent field investigations indicated that malaria active case detection practices (including RACD) varied widely from county to county [15]. Therefore, an assessment of the ‘1-3-7’ performance in the Yunnan Province border region was conducted to identify the gaps and challenges of the system with a focus on RACD-related activities. The ways to optimize the response strategy to the local Yunnan setting were explored to promote malaria elimination in the study areas.

## Methods

### Study areas

Yunnan Province—situated in the southwestern part of China with an area of 394,000 km^2^ and 4061 km of borderline—has a population of 45 million. The rural population along this border area is among the poorest in China with an average annual gross domestic product per capita of <US$ 100 [16]. Historically, Yunnan Province has stable malaria endemicity due to its mountainous valleys, proximity to the Indian Ocean and a Pacific monsoon climate along with frequent human movement contributing to the highest burden of malaria in the areas that border Myanmar. The malaria cases detected in 18 counties along the China–Myanmar border account for about 30% of the total cases in China, with more than 95% of them in the border region being imported. The border areas face many challenges including continuous importation of malaria infections, increases in population movement and wide distribution of efficient vectors [17].

### Study sites

**Fig. 1:**
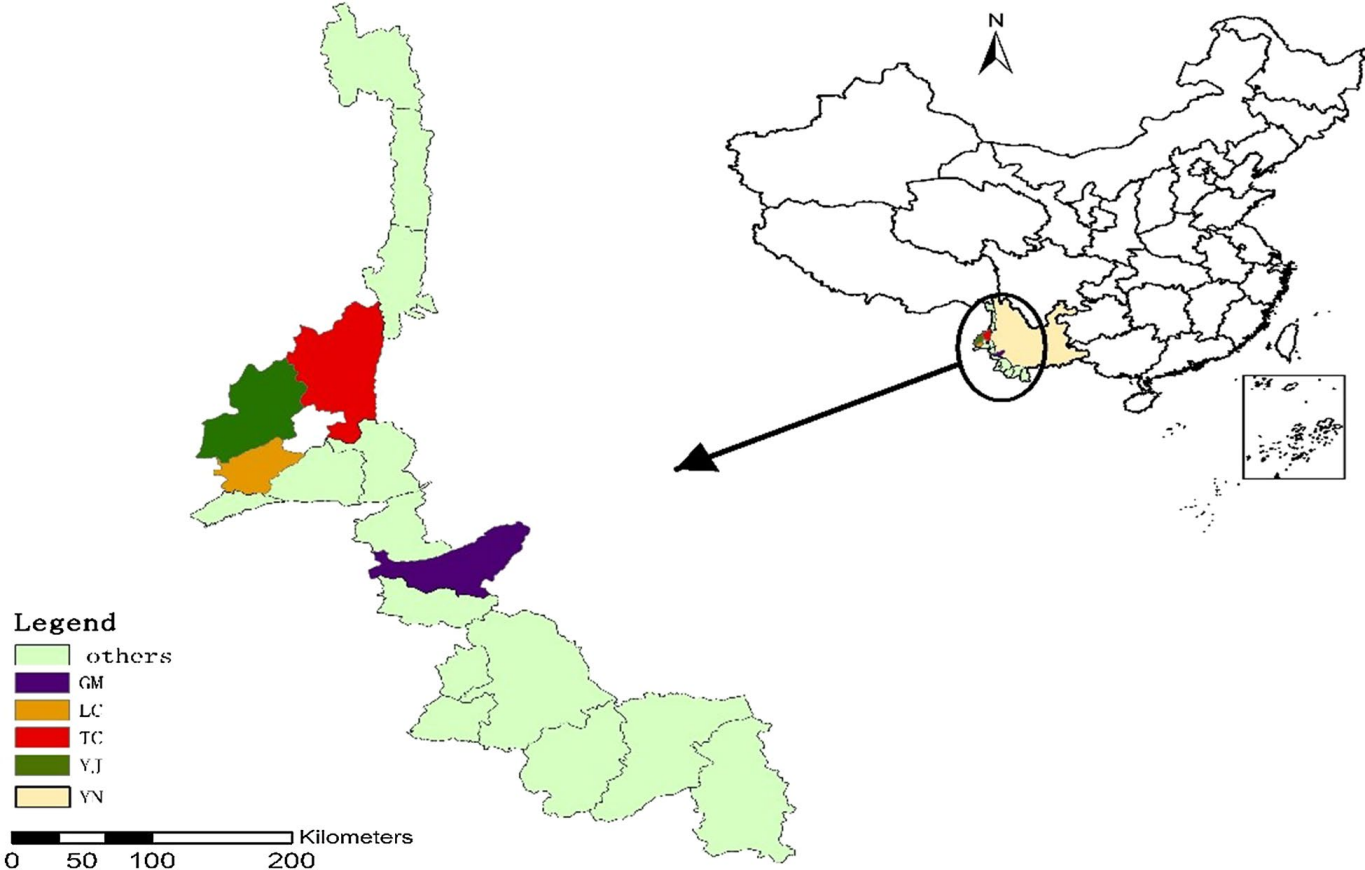
Map of study area; China, Yunnan province, study counties

Sampled counties were chosen based on having the highest malaria incidence in Yunnan Province from 1 January 2012 to 31 December 2014. A total of four counties (Genma, Longchuan, Tengchong, Yingjiang) out of 18 total border counties were identified with three townships randomly selected from each of the sampled counties as the study sites (Fig. 1). There are 16 health facilities (including 4 County CDCs and 12 township hospitals) in the study areas that are required to report online all suspected and laboratory-confirmed malaria cases through the China Information System for Disease Control and Prevention (CISDCP).

### Study design

A mixed methods retrospective study including the quantitative and qualitative survey and analysis of the 1-3-7 system components was conducted.

### Study population

Key health facility personnel from 4 local county CDCs and 12 township hospitals having at least 3 years of experiences on malaria control and elimination were selected from each sampled study area to assess the knowledge as well as practices of malaria case reporting and follow-up.

### Study procedures

Questionnaires were administered to the sampled township hospitals and county CDC personnel involved in case reporting, case investigation and RACD activities. A total of 29 individuals were administered self-reported questionnaires from May to July 2015 using the standardized questionnaires with open- and closed-questions developed based on the local standard operating procedures. Questions were designed to assess the knowledge and practices of malaria case reporting, case investigation and RACD for key personnel conducting these activities.

Data on malaria case reporting, case investigation and RACD were extracted from the patients registered in 4 local CDCs and 12 sampled township hospitals for the 3-year period from 1 January 2012 to 31 December 2014. Data on malaria case reporting were extracted from the CISDCP database of the sampled counties. Data were queried and confirmed between May and December, 2015. Variables assessed in this review included the date of diagnosis, date of case reporting, date of case investigation, case classification (local *vs* imported), date of RACD, population tested living in areas during RACD and number of individuals tested during RACD.

### Quality control and assurance

The study protocol was developed and finalized with inputs from stakeholders as well as experts. A pre-survey was conducted and the questionnaire was finalized before start of the study. Data were collected using Microsoft Excel and data were double entered. All data were summarized and verified with the sampled township hospitals and local CDCs within 15 days after completion of field survey.

### Data analysis

Summary statistics and proportions were used to compare case reporting completeness, and timeliness based on the CISDCP database and original patient registers in the study areas. Case investigation and RACD screening summary statistics and proportions were based on the original patient registers among the 4 sampled counties. RACD indicators were compared between the local and imported cases, high and low transmission seasons. RACD completion rates were further analysed using Stata software, version 13.1 (Stata Corporation, College Station, TX, USA). Chi square tests (χ^2^) were used. Confidence intervals are 95% and P values are two tailed. The indicator for ‘population living in areas tested’ is the estimated population residing in a natural village where those individuals are at risk of contracting malaria. Malaria cases were defined based on the diagnostic criteria of World Health Organization (WHO). Annual malaria incidence was calculated by using the annual population of each county. Demographic data at the county level was obtained from the Chinese Natural Resources Database [18].

## Results

A total of 29 key health facility staff were interviewed about their knowledge of case investigation and RACD activities in the study areas. Malaria case reporting, investigation and RACD indicators were assessed and results described below.

### Knowledge of case investigation and RACD

Sixty-nine percent (20/29) and 65% (19/29) of respondents correctly stated that case investigation and RACD should occur in 3 and 7 days, respectively (Table 1). 76% (22/29) of respondents stated that all household members (asymptomatic and fever) should be tested during RACD but only 42% (13/31) stated that testing be conducted by visiting each individual household. The knowledge of the minimum geographic radius to test around an index case household varied greatly from 50 to 100 m (38%, 11/29) up to 1 km (10%, 3/29).

**Table 1:** Summary of case investigation and RACD knowledge and practices

Question 1	Within how many days should case investigation occur?		
	Answer	n	%
	1 day	2	7
	2 days	2	7
	3 days	20	69
	7 days	5	17
	Total	29	100

Question 2	Within how many days should RACD occur?		
	Answer	n	%
	1 day	2	7
	3 days	6	21
	4–5 days	2	7
	7 days	19	65
	Total	29	100

Question 3	What is the minimum geographic radius to screen around an index case household during RACD?		
	Answer	n	%
	<50 m	8	28
	50–100 m	11	38
	>100–500 m	7	24
	>500–1000 m	3	10
	Total	29	100

Question 4	Which individuals should you screen when conducting RACD around an index case household?		
	Answer	n	%
	Febrile only	6	21
	All (asymptomatic and febrile)	22	76
	Other	1	3
	Total	29	100

Question 5	How do you conduct RACD around the index case household?		
	Answer	n	%
	Visit each house and test household members	13	42
	Collect the community members into one location and test them	18	58
	Total	31	100

Question 6	What is the minimum geographic radius to screen around an index case household during RACD?		
	Answer	n	%
	<50 m	8	28
	50–100 m	11	38
	>100–500 m	7	24
	>500–1000 m	3	10
	Total	29	100

### Summary of case reporting and investigations

A total of 260 cases were recorded at the sampled township hospitals and county CDCs from 2012 to 2014 (Table 2). All cases were reported to the CISDCP database and within 1 day of presentation to the health facilities. All 260 cases had completed case investigations. The timeliness of case investigations conducted within 3 days was 81.5% (212/260) across the all sampled areas.

**Table 2:** Total case reporting and case investigation timeliness in study areas, 2012-2014

Study county	Cases recorded (% of total)	Cases reported			Cases investigated		
		No.	Within 1 day	%	No.	Within 3 days	%
GM	31 (11.9)	31	31	100	31	24	77.4
LC	27 (10.4)	27	27	100	27	24	88.9
TC	146 (56.2)	146	146	100	146	118	80.8
YJ	56 (21.5)	56	56	100	56	46	82.1
Total	260	260	260	100	260	212	81.5

### Summary of RACD

#### Screening during high and low transmission seasons

Of the 260 malaria cases that were reported and investigated, 70.0% (182/260) had RACD screening conducted and 90.1% (164/182) of the RACD screenings occurred within 7 days (Table 3). Differences were observed in reported cases that had RACD follow-up between the high (May–October) and low (November–April) transmission seasons 86.7% (130/150) and 47.3% (52/110), respectively (χ^2^ = 46.90, p < 0.000). RACD event follow-up within 7 days showed similar timelines with 89.2% (116/130) and 94.1% (48/51) in the high and low transmission seasons, respectively.

**Table 3:** Comparison of RACD reporting and timeliness in high and low transmission seasons in study areas, 2012–2014

Study county	High transmission season (May to Oct)			Low transmission season (Nov to April)			Total RACD events occurred (%)	Total RACD events occurred within 7 days (%)
	Reported cases (% of total)	RACD events occurred (%)	RACD events occurred within 7 days (%)	Reported cases (% of total)	RACD events occurred (%)	RACD events occurred within 7 days (%)		
GM	18 (12.0)	17 (94.4)	14 (82.4)	13 (12)	4 (30.8)	4 (100.0)	21	18
LC	18 (12.0)	17 (94.4)	16 (94.1)	9 (8)	3 (33.3)	2 (66.7)	20	18
TC	69 (46.0)	55 (79.7)	50 (90.9)	77 (70)	41 (53.3)	39 (95.1)	96	89
YJ	45 (30.0)	41 (91.1)	36 (87.8)	11 (10)	4 (36.4)	3 (75.0)	45	39
Total	150 (58)	130 (86.7)	116 (89.2)	110 (42)	52 (47.3)	48 (92.3)	182 (70.0%)	164 (90.1%)

Pearson’s χ^2^ = 46.90, p < 0.000

## RACD results

Differences in RACD screening results were identified when comparing local and imported index cases in the high and low transmission seasons (Table 4). Of the 182 cases that had RACD completed, 71% (130/182) were conducted during the high transmission seasons, with 100% (39/39) of local and 82% (91/111) of imported cases completed. In both high and low transmission seasons, 89% (41/46) of local cases had RACD conducted in 7 days. Follow-up of imported cases within 7 days during the high transmission seasons was 86% (78/91).

**Table 4:** RACD comparison by local and imported in high and low transmission seasons, 2012–2014

Season and case classification	Cases reported	RACD events completed		RACD events occurred within 7 days		Population living in areas tested	Population tested for malaria		Positive cases		Average number individuals tested per RACD
	n	n	%	n	%	n	n	%	n	%	
High transmission + local	39	39	100.0	34	87.2	3318	508	15.3	4	0.79	13.0
High transmission + imported	111	91	82.0	78	85.7	17,434	2122	12.2	6	0.28	23.3
Low transmission + local	7	7	100.0	7	100.0	648	83	12.8	0	–	11.9
Low transmission + imported	103	45	43.7	45	100.0	10,008	949	9.5	0	–	21.1
Total	260	182	70.0	164	90.1	31,408	3662	11.7	10	0.27	17.3

Of the 31,408 individuals that live in villages where the 182 RACD events took place, 11.7% (3662/31,048) were tested for malaria using rapid diagnostic test (RDT) or microscopy (Table 4). After local index cases were identified during high transmission seasons, 15.3% (508/3318) were tested compared to 12.2% (2122/17,434) following imported index case identification. The average number of individuals tested per RACD event completed was the highest with imported index cases during high (23.3%) and low transmission (21.1%) seasons. All malaria-positive individuals (10 total) identified during RACD were in the high transmission seasons with 40% (4/10) and 60% (6/10) from local and imported cases, respectively.

## Discussion

Although study results showed that malaria case reporting was 100%, critical gaps were identified in the timeliness and completeness of case investigation and RACD follow-up activities, as well as the knowledge and practices of malaria personnel conducting response activities.

Results for 2012–2014 in the study areas showed that malaria case reporting and case investigation was 100% complete. This is possible due to China enacting legislation mandating that all suspected or confirmed malaria cases are reported within 1 day of presentation for diagnosis [1]. Adherence to mandatory reporting standards is supported by a reliable web-based reporting system through the CISDCP which enables rapid reporting within 1 day. However, the timeliness of the case investigations conducted within 3 days was only 81.5%, because it is very difficult for timely follow-up by County CDC staff in the remote and mountainous terrain or border areas with highly mobile populations [17, 19], and local County CDC staff may not be available to conduct the malaria case investigations due to having other responsibilities. In these circumstances, staff in county hospitals would be required to undertake the case investigations. This poses a challenge because health facility staff may not have been trained on malaria case investigations. Therefore, training of county hospital staff on case investigation and foci treatment may alleviate this challenge when County CDC staff are not available.

Study findings also indicated that there was a lack of standard operating procedures (SOPs) and, therefore, knowledge on the ‘1-3-7’ strategy targets, highlighted in how different activities were carried out. Though a national action plan for malaria elimination is available [1], SOPs for response activities do not exist. For example, less than half of respondents (42%) stated that RACD around an index case household should be conducted by visiting individual households rather than community screening in a single location. This may lead to unsatisfactory coverage of the targeted population, with only those who are living nearby or interested in being tested for malaria actually receiving a test. Additionally, 21% of respondents stated that they only test the symptomatic individuals during RACD events instead of all individuals residing in a pre-determined radius around a local index case. A review of Asia Pacific countries showed a wide variety of screening procedures for RACD indicating that gaps remain in the optimal methods to identify additional malaria infections during RACD [6]. However, given the importance and frequency of asymptomatic infections in low transmission settings, testing symptomatic individuals alone will not effectively reduce the residual transmission [20, 21]. The NMEP should create detailed SOPs for RACD and foci response, including testing of all individuals living in an area with a recent index case to achieve higher coverage.

Although, the NMEAP describes that all imported and local index cases should have RACD completed within 7 days when transmission risk is believed to be possible [22], RACD response was lacking in both completeness and timeliness; only 70% of malaria cases had RACD follow-up conducted during the study period. In addition, more evidence is required to determine the most effective diagnostic tools for RACD screening practices in elimination settings. RACD activities in the study areas relied heavily on rapid diagnostic tests (RDTs), yielding only 10 additional positive cases. The total positivity rate of 0.27% is lower than that of national sentinel surveillance results with a positivity rate of 0.8% in 2013 [23], highlighting the inefficiency of identifying additional cases with RACD activities in the border area. Meanwhile, four infections were found from a cohort of 505 RDTs (0.8%) and microscopy-negative people in the same areas in 2013 using CLIP-PCR.

All 10 additional infections identified in this study were in the high transmission seasons; 4 infections (0.79% positivity) found with RACD around suspected local cases and 6 infections (0.28% positivity) around suspected imported cases. Since only household and community members with fever were tested using RDTs during RACD, the detection of very low density infections is critically important for China in its pursuit of elimination. The availability of highly sensitive and field-friendly RDT or other molecular technique such as LAMP [23] or PCR [24] would be ideal in this border region. However, in the absence of this, presumptive treatment during the high transmission seasons targeting high-risk individuals may be a strategy to address the challenge of low density infections missed by RDTs and poor RACD screening coverage [25].

Despite China’s efforts to strengthen intervention activities in the border region [26], residual malaria transmission continues to be a challenge. Recent evidence indicates that malaria importation and local vector susceptibility to imported *Plasmodium vivax* infections remains a continued risk along the China–Myanmar border [27]. Difficulty in classifying malaria cases as local or imported in highly mobile populations residing along the border area is also a challenge in determining the risk of resurgence from imported cases [28]. Improving the case investigation questionnaire to obtain a detailed travel history and risk behaviours may help improve case classifications as imported or local. The combination of more accurate information and developing maps for foci of transmission by geo-locating cases to the household can support the targeting of these high-risk individuals and their communities with malaria interventions [29, 30]. Furthermore, developing local capacity for genotyping of all local and imported malaria cases would be an important tool for Yunnan to determine the relatedness of malaria infections and distinguish between local and imported malaria [31].

The Yunnan border region is the ideal environment for malaria transmission. Political instability, military conflict, and a fragile health system in Myanmar, coupled with population movement along the shared border, make malaria control activities in this porous region challenging. Greater economic and health care support has been recently proposed by the Chinese government to cooperate with neighboring countries [32]. This cross-border support was also recently highlighted in the Global Technical Strategy for Malaria 2016–2030 [33] and the Strategy for Malaria Elimination in Greater Mekong Subregion (2015–2030) [34] to enable good commitment, collaboration and coordination. As the threat of multidrug resistance looms in the Greater Mekong Subregion, effective cross-border collaboration through a multi-sectoral approach is essential for both countries to support malaria elimination.

Operational research specific to this border region is also needed to support evidence-based strategies due to the unique challenges in Yunnan Province, including hard-to-reach, mobile populations, and a variety of different mosquito vectors. For example, an understanding of when to initiate the screening response after an imported case is identified—whether 7 days or possibly longer (i.e., 2–3 weeks)—is needed. By tailoring the response to this local context, available resources can be maximized to promote and accelerate malaria elimination in the border region.

Study limitations include the potential bias associated with records from public health facilities. Bias or error may exist between the log records or working reports and practices of the staff responsible for completing the reports. The authors did not explore the reasons why RACD was unable to be completed in areas within study counties; however, a deeper understanding of these reasons may help to identify ways to alleviate barriers to follow-up (Additional files 1 and 2).

## Conclusions

Study results identified gaps in the knowledge and practices of RACD activities. The ‘1-3-7’ strategy would benefit by considering malaria intervention responses adapted to the unique and challenging setting of mountainous border regions. Improving the knowledge and training of all staff responsible for RACD will support the standardization and implementation of response activities to promote malaria elimination in China.

## Additional files

**Additional file 1.** Questionnaire responses.

**Additional file 2.** Questionnaires.

## Abbreviations

RACD: reactive case detection
NMEP: National Malaria Elimination Programme
NMEAP: National Malaria Elimination Action Plan
CISDCP: China Information System for Disease Control and Prevention
CDC: Center for Disease Control and Prevention
WHO: World Health Organization
SOP: standard operating procedure
RDT: rapid diagnostic test
LAMP: loop-mediated isothermal amplification
PCR: polymerase chain reaction
