# Supplementary material for: Adapting the local response for malaria elimination through evaluation of the 1-3-7 system performance in the China–Myanmar border region
Source: Malar J. 2017 Jan 31;16:54. doi: 10.1186/s12936-017-1707-1 (PMC5282924; doi:10.1186/s12936-017-1707-1)
Supplement: Supplementary file 1 — Additional file 1. Questionnaire responses. [file 12936_2017_1707_MOESM1_ESM.docx]

**Table S1. Summary of the questionnaire for cases investigation of local health staff**

| **1** | | | **2** | | | **3** | | | **4** | | | **5** | | | **6** | | | **7** | | |
| --- | --- | --- | --- | --- | --- | --- | --- | --- | --- | --- | --- | --- | --- | --- | --- | --- | --- | --- | --- | --- |
| Answer | No. | % | Answer | No. | % | Answer | No. | % | Answer | No. | % | Answer | No. | % | Answer | No. | % | Answer | No. | % |
| 1 | 25 | 86.2 | 1 day | 2 | 6.9 | 1 | 8 | 27.6 | 1 | 10 | 34.5 | 1 | 18 | 62.1 | 1 | 18 | 62.1 | 1 | 2 | 6.9 |
| 2 | 2 | 6.9 | 2 days | 2 | 6.9 | 2 | 26 | 89.7 | 2 | 0 | 0.0 | 2 | 0 | 0.0 | 2 | 0 | 0.0 | 2 | 8 | 27.6 |
| 3 | 4 | 13.8 | 3 days | 20 | 69.0 | 3 | 0 | 0.0 | 3 | 1 | 3.4 | 3 | 0 | 0.0 | 3 | 6 | 20.7 | 3 | 8 | 27.6 |
| 4 | 0 | 0.0 | 7 days | 5 | 17.2 | 4 | 0 | 0.0 | 4 | 21 | 72.4 | 4 | 1 | 3.4 | 4 | 8 | 27.6 | 4 | 1 | 3.4 |
|  |  |  |  |  |  |  |  |  | 5 | 10 | 34.5 | 5 | 1 | 3.4 | 5 | 2 | 6.9 | 5 | 0 | 0.0 |
|  |  |  |  |  |  |  |  |  | 6 | 0 | 0.0 | 6 | 7 | 24.1 |  |  |  | 6 | 6 | 20.7 |
|  |  |  |  |  |  |  |  |  | 7 | 0 | 0.0 | 7 | 3 | 10.3 |  |  |  | 7 | 4 | 13.8 |
|  |  |  |  |  |  |  |  |  |  |  |  | Others | 0 | 0.0 |  |  |  | 8 | 0 | 0.0 |
|  |  |  |  |  |  |  |  |  |  |  |  |  |  |  |  |  |  | 9 | 7 | 24.1 |
|  |  |  |  |  |  |  |  |  |  |  |  |  |  |  |  |  |  | 10 | 1 | 3.4 |
|  |  |  |  |  |  |  |  |  |  |  |  |  |  |  |  |  |  | 11 | 0 | 0.0 |

**Table S2. Summary of the questionnaire for reactive case detection (RACD) of local health staff**

| **1** | | | **2** | | | **3** | | | **4** | | | **5** | | | **6** | | | **7** | | | **8** | | | **9** | | | **10** | | | **11** | | |
| --- | --- | --- | --- | --- | --- | --- | --- | --- | --- | --- | --- | --- | --- | --- | --- | --- | --- | --- | --- | --- | --- | --- | --- | --- | --- | --- | --- | --- | --- | --- | --- | --- |
| An | No | % | An | No | % | An | No | % | An | No | % | An | No | % | An | No | % | An | No | % | An | No | % | An | No | % | An | No | % | An | No | % |
| 1 | 18 | 62 | 1day | 2 | 7 | 10pop | 3 | 10 | 4 | 5 | 17 | 10 | 1 | 3 | 1 | 6 | 21 | 1 | 0 | 0 | 1 | 13 | 45 | 1 | 21 | 72 | 1 | 0 | 0 | 1 | 19 | 66 |
| 2 | 1 | 3 | 3day | 6 | 21 | 12pop | 2 | 7 | 5 | 8 | 28 | 15 | 1 | 3 | 2 | 22 | 76 | 2 | 14 | 48 | 2 | 18 | 62 | 2 | 1 | 3 | 2 | 20 | 69 | 2 | 18 | 62 |
| 3 | 9 | 31 | 4day | 1 | 3 | 15pop | 10 | 34 | 8 | 1 | 3 | 20 | 1 | 3 | 3 | 1 | 3 | 3 | 24 | 83 |  |  |  | 3 | 0 | 0 | 3 | 19 | 66 | 3 | 14 | 48 |
| 4 | 17 | 59 | 5day | 1 | 3 | 20pop | 5 | 17 | 10 | 2 | 7 | 25 | 2 | 7 |  |  |  | 4 | 0 | 0 |  |  |  | 4 | 0 | 0 | 4 | 0 | 0 | 4 | 10 | 34 |
|  |  |  | 7day | 19 | 66 | 50pop | 2 | 7 | 15 | 4 | 14 | 30 | 3 | 10 |  |  |  | 5 | 1 | 3 |  |  |  | 5 | 0 | 0 | 5 | 0 | 0 | 5 | 15 | 52 |
|  |  |  |  |  |  | 150pop | 1 | 3 | 18 | 1 | 3 | 50 | 9 | 31 |  |  |  |  |  |  |  |  |  | 6 | 10 | 34 |  |  |  | 6 | 27 | 93 |
|  |  |  |  |  |  | 10% | 5 | 17 | 20 | 3 | 10 | 75 | 1 | 3 |  |  |  |  |  |  |  |  |  | 7 | 0 | 0 |  |  |  | 7 | 0 | 0 |
|  |  |  |  |  |  | 90% | 1 | 3 | 25 | 1 | 3 | 100 | 1 | 3 |  |  |  |  |  |  |  |  |  | 8 | 0 | 0 |  |  |  |  |  |  |
|  |  |  |  |  |  |  |  |  | 10% | 3 | 10 | 300 | 1 | 3 |  |  |  |  |  |  |  |  |  |  |  |  |  |  |  |  |  |  |
|  |  |  |  |  |  |  |  |  | 90% | 1 | 3 | 400 | 2 | 7 |  |  |  |  |  |  |  |  |  |  |  |  |  |  |  |  |  |  |
|  |  |  |  |  |  |  |  |  |  |  |  | 500 | 4 | 14 |  |  |  |  |  |  |  |  |  |  |  |  |  |  |  |  |  |  |
|  |  |  |  |  |  |  |  |  |  |  |  | 1000 | 3 | 10 |  |  |  |  |  |  |  |  |  |  |  |  |  |  |  |  |  |  |
