# Supplementary material for: Adapting the local response for malaria elimination through evaluation of the 1-3-7 system performance in the China–Myanmar border region
Source: Malar J. 2017 Jan 31;16:54. doi: 10.1186/s12936-017-1707-1 (PMC5282924; doi:10.1186/s12936-017-1707-1)
Supplement: Supplementary file 2 — Additional file 2. Questionnaires. [file 12936_2017_1707_MOESM2_ESM.docx]

**Questionnaire for Case Investigation and Reactive Case Detection Survey Evaluation**

ID:___________________ Position:_____________________ Health Facility:____________________

***Attention****: Complete the section(s) below that is relevant to your current job responsibilities:*

1. Case Investigation: Complete **Section 1 only**

2. Reactive Case Detection: Complete **Section 2 only**

3. Both Case Investigation and Reactive Case Detection: Complete **both Sections 1 and 2**

**Section 1. Case Investigation**

1. What is the policy for conducting a case investigation? (knowledge)
   1. All indigenous and imported cases
   2. Indigenous cases only
   3. Imported cases only
   4. Other – Please specify:
2. Within how many days should the positive case be investigated? (knowledge) _____________

*[cross check individual level data to get mean/range times]*

1. When doing a case investigation in the village, which of the following best describes how you make an appointment with that case? (Circle all that apply) (practice)
   1. Telephone the case
   2. No communication - go to the case residence to see if he or she is home
   3. Inform volunteers to make appointment with the case
   4. Other – Please specify:
2. What do you do the most if the case is not home when you visit? (practice)
   1. We do not re-visit the index case
   2. We mark the case as imported
   3. We mark the case as “not found”
   4. Visit a second time: later that day or on a subsequent day
   5. Telephone to schedule an appointment
   6. Inform volunteers to make appointment with the case
   7. Other – Please specify:
3. What time of day do you normally investigate cases? (Circle all that apply) (practice)
   1. Right after case detected at malaria clinics
   2. Before 8am
   3. Between 8am-12noon
   4. Between 12noon-5pm
   5. After 5pm
   6. Weekends
   7. Whenever the team is available
   8. Other – Please specify:
4. What information do you use to determine if the malaria case is local? (knowledge)
   1. Case has travel history to malaria endemic areas outside of local county
   2. Case traveled outside county within the last 2 weeks (but not within the last week)
   3. Presence of malaria vector
   4. The case stays in the village within the last two weeks
   5. Other – Please specify:
5. If any case is not investigated, what are the main reasons these cases are not investigated?

(Circle all that apply) (practice)

- 1. It is an imported case/migrant
  2. It is outside of my district
  3. The person could not be found
  4. Not enough staff/resources
  5. Case was in a remote area and unable to access
  6. Daily cross border case
  7. Case was notified too late
  8. Case was detected in other health facilities
  9. Not applicable – we investigate every case
  10. Incomplete patient Information
  11. Other – Please specify:

1. What are some of the challenges in conducting case investigation?

…………………………………………………………………………………………………………………………………..……………………………………………………………………………………

……

**Section 2. Reactive Case Detection**

1. What triggers screening in the community? (knowledge)
   1. All cases in endemic villages
   2. All positive cases
   3. New foci
   4. All cases in village with presence of vector
2. Within how many days should reactive case detection occur?___________________
   (knowledge) *[cross check individual level data to get mean/range times]*
3. What is the minimum number of **people** to be screened around a positive case? (knowledge)

Number of people screened:___________________

1. What is a minimum number of **households** to be screened around a positive case? (knowledge)

Number of households screened:___________________

1. What is a minimum **geographic radius** to be screened around a positive case? (knowledge)

Number of meters radius screened:_____________________

1. When screening household members do you screen: (knowledge)
   1. Febrile cases only
   2. All household members (asymptomatic and febrile cases)
   3. We do not screen household members of a positive case
2. What do you do if someone from the household of the malaria case is not home and you cannot screen them? (Circle all that apply) (practice)
   1. We do not re-visit household members
   2. Visit a second time: later that day or on a subsequent day
   3. Telephone to schedule an appointment
   4. Inform volunteers to make appointment with the household members
   5. Other – Please specify:
3. When screening neighbors of the index case household, do you: (practice and knowledge)
   1. Visit each house and screen only those household members.

Why?__________________________

- 1. Collect the community members in to one location and screen there.

Why?__________________________

1. What time of day do you normally conduct screening in the community? (practice)

(Circle all that apply)

- 1. Right after case detected at malaria clinics
  2. Before 8am
  3. Between 8am-12noon
  4. Between 12noon-5pm
  5. After 5pm
  6. Weekends
  7. Whenever the team is available
  8. Other – Please specify:

1. If an individual is missing at the time of screening in the community, what do you do? (Circle all that apply) (practice)
   1. We do not re-visit the individual
   2. Visit a second time: later that day or on a subsequent day
   3. Telephone the individual to schedule an appointment
   4. Inform volunteers to make appointment with the individual
   5. Other – Please specify:
2. What other activities do you do during reactive case detection? (practice)
   1. Indoor residual spraying
   2. LLIN distribution/ ITN
   3. Mosquito species identification/ entomological survey
   4. Larviciding
   5. Case finding
   6. Health promotion
   7. Other activities; Please specify:
3. What are some of the challenges in conducting screening in the community?

…………………………………………………………………………………………………………………………………..…………………………………………………………………………………………………………………………………..…………………………………
